# Adherence to COVID-19 vaccination in riverside populations of the municipality of Careiro/AM

**DOI:** 10.1590/0034-7167-2025-0066

**Published:** 2026-08-07

**Authors:** Hibelfran Alfaia Damasceno, Vivian Cristine de Souza e Souza, Luiz Henrique Gonçalves Maciel, Marcel Gonçalves Maciel

**Affiliations:** I Universidade Federal do Amazonas. Manaus, Amazonas, Brazil; II Universidade do Estado do Amazonas. Manaus, Amazonas, Brazil

**Keywords:** Vaccination, Immunization Schedule, COVID-19, Rural Population, Rural Spatial Distribution., Vacunación, Esquemas de Inmunización, COVID-19, Población Rural, Población Ribereña.

## Abstract

**Objectives:**

to describe adherence to the COVID-19 vaccination schedule in two riverside communities in Careiro/AM, Brazil.

**Methods:**

this was an epidemiological, observational, descriptive, and cross-sectional study with a quantitative approach, conducted in 2023-2024. Data were collected via interviews guided by a standardized form and analyzed descriptively in Excel (relative and absolute values).

**Results:**

sociodemographic characteristics were similar to those found in the literature. The majority received at least one dose (98.68%). Access, concerns, and social factors influenced vaccination. Unwillingness to be vaccinated predominated (36.84%). Thus, 62.50% were identified as having no comorbidities and 56.29% had a reported diagnosis of COVID-19.

**Conclusions:**

the riverside communities surveyed are undergoing transformations. Furthermore, they exhibit a progressive refusal of vaccine doses, which appears to be associated with social factors and information about the vaccines.

## RESUMEN

**Objetivos:**

describir la adherencia al esquema de vacunación contra la COVID-19 en dos comunidades ribereñas de Careiro/AM.

**Métodos:**

estudio epidemiológico, observacional, descriptivo y transversal con enfoque cuantitativo, realizado entre 2023 y 2024. Los datos se recopilaron mediante entrevistas, guiadas por un formulario estandarizado, y se analizaron descriptivamente en Excel (valores relativos y absolutos).

**Resultados:**

las características sociodemográficas fueron similares a las descritas en la literatura. La mayoría recibió al menos una dosis (98,68%). El acceso, la preocupación y factores sociales influyeron en la vacunación. Predominó la reticencia a vacunarse (36,84%). El 62,50% no presentaba comorbilidades y el 56,29% tenía un diagnóstico de COVID-19.

**Conclusiones:**

las comunidades ribereñas estudiadas están experimentando transformaciones. Además, muestran un rechazo progresivo a la vacunación, lo que parece estar asociado a factores sociales y a la falta de información sobre las vacunas.

## INTRODUCTION

SARS-CoV-2 (Severe Acute Respiratory Syndrome Coronavirus 2), responsible for the COVID-19 pandemic, causes infectious respiratory disease and affects anyone, manifesting in mild or severe forms^(1)^. Transmission, which occurs through the respiratory route, via droplets or aerosols, is related to high transmissibility^(2)^. In this context, several global initiatives were developed and implemented to contain the pandemic.

Vaccines, one of the initiatives launched in 2020, represent indispensable tools in the fight against COVID-19, as they guarantee individual and collective prevention of severe forms of the disease by sensitizing the body’s immune system to recognize and create antibodies against the virus. However, they can cause mild or severe side effects, which tend to disappear within a few days^(3,4)^. Immunization is essential in controlling the spread of the COVID-19 pandemic^(5)^.

The World Health Organization led the COVID-19 Vaccines Global Access Center (COVAX Facility initiative), which optimized the timeline for vaccine production and licensing^(6)^. Furthermore, it played a leading role in developing a toolkit with guidelines, training, and information to support vaccine distribution programs. Alongside these efforts, randomized controlled trials were conducted to verify vaccine safety and efficacy^(7)^.

In Brazil, although vaccination began in 2021 using vaccines authorized for emergency use, it was in 2022 that the first National Operational Plan for Vaccination against COVID-19 was launched^(8,9)^, updated in 2023^(10,11)^. Starting in June 2024, a new vaccination guideline was introduced in Brazil using the monovalent XBB vaccine, manufactured by *Moderna*. The guidelines considered different age groups and priority groups, such as riverside communities^(12)^.

However, challenges persist, such as vaccine hesitancy, manifesting as individuals’ resistance to accepting vaccines, even when they are safe, effective, and accessible for disease prevention^(13,14)^. For riverside communities, which have specific social determinants, vaccination efforts must consider economic and logistical challenges, since in more remote areas, staggered vaccinations do not present a positive cost-benefit ratio and run the risk of introducing the virus^(11)^.

The riverside peoples are located on the banks of “streams, flooded forests, lakes, and floodplains”^(15)^, and are classified as part of non-indigenous traditional peoples, who emerged from colonial and migratory miscegenation^(16,17)^. The combination of unfavorable economic conditions, climate change, and geographical limitations represents barriers to access to healthcare in these communities. Furthermore, riverside dwellers have a unique lifestyle, with collective work and social behaviors strongly based on their family and community structure, access to which requires specialized logistics^(18)^.

COVID-19 is a highly transmissible disease that, in a short period of time, has already shown genetic mutations in the SARS-CoV-2 virus^(19)^. In Brazil, there was a high spread of the disease, evidenced by the number of new cases. By 2024, in the Northern region, more than 2 million confirmed cases and more than 52,000 deaths had been recorded^(20)^. The state of Amazonas, in turn, accumulated 643,730 cases and 14,528 deaths. The capital, Manaus, with more than 2 million inhabitants, concentrated more than 190,000 cases and 9,000 deaths. The municipality of Careiro, in the Metropolitan Region of Manaus, with a population of 37,869 inhabitants (Brazilian Institute of Geography and Statistics, 2022), recorded 6,900 confirmed cases and 93 deaths^(21)^.

Given this scenario, COVID-19 vaccines have been an indispensable tool in protecting against and controlling the pandemic. However, in the municipality of Careiro, which has a significant rural population, including riverside communities, state records indicate a drop in vaccination coverage between the first and second doses, reaching 69.4% and 58.5%, respectively. Booster rates showed an even sharper reduction, with the first booster reaching 34.5%, and the second, 12.9%. Additionally, the primary vaccination schedule reached only 61% of the total population^(21,22)^. Considering the information presented about the COVID-19 pandemic and the specific characteristics of traditional riverside communities, this project aimed to answer the following question: how does adherence to the COVID-19 vaccination schedule occur in two traditional riverside communities in the municipality of Careiro/AM?

## Study relevance

This manuscript is an excerpt from the methodological study originating from the dissertation^(23)^ entitled “*Vacinas covid-19: adesão ao esquema vacinal em comunidades ribeirinhas do município do Careiro/AM*”.

## OBJECTIVES

To describe adherence to the COVID-19 vaccination schedule in two riverside communities in the municipality of Careiro/AM.

## METHODS

### Ethical aspects

The study was conducted in accordance with national and international ethical guidelines, as per Resolution 466/12, and was approved by the *Universidade Federal do Amazonas* Research Ethics Committee (REC), as per the Certificate of Presentation of Ethical Consideration 71215323.4.0000.5020. Data collection began after the project was approved by the aforementioned REC. All participants were required to obtain written Informed Consent Form (ICF).

### Study design, period and location

It is outlined within an epidemiological, observational, descriptive, and cross-sectional study, with a quantitative approach^(24,25)^. The research followed the EQUATOR network STrengthening the Reporting of OBservational studies in Epidemiology. The research was carried out in one of the 39 communities^(26)^, belonging to the municipality of Careiro (Tilheiro and Samaúma), conducted in months of 2023 and 2024, through in-person visits. Careiro is a municipality in Amazonas that belongs to the Metropolitan Region of Manaus. According to municipal indicators, most of the population resides in the rural area (23,297 people)^(27)^. The communities selected to comprise the study area and population are Tilheiro and Sumaúma, which have the typical characteristics of traditional riverside communities^(28,29)^.

### Population and sample

The approach was carried out sequentially during home visits to research participants, selecting one out of every five households. Given the peculiar aspects of the communities studied, which include difficult access due to geographical location and transportation conditions, further complicated by the seasonal cycles typical of the Amazon region, a non-probabilistic convenience sample was used, in which research elements are selected according to the convenience or ease for the researcher^(30)^.

### Inclusion and exclusion criteria

Individuals aged 18 years or older, residing in the study area, and capable of understanding the interview questions (as assessed by the interviewer’s perception) were included. Individuals with specific contraindications to the COVID-19 vaccine (hypersensitivity to the vaccine’s active ingredients), mental disability (reported by a responsible adult present), communication difficulties (as assessed by the interviewer’s perception), unfavorable health conditions at the time of the visit (acute or severe symptoms, impaired state of consciousness/cognition, as assessed by the interviewer’s perception), under 18 years of age, and those unable to understand the interview questions (as assessed by the interviewer’s perception) were excluded.

### Study protocol

Data collection was conducted through interviews guided by a standardized questionnaire to obtain socioeconomic, clinical, and behavioral information regarding COVID-19 vaccination. In-person visits were carried out in the communities of Tilheiro and Samaúma. The approach occurred randomly at residents’ homes, where the research objectives were explained. Following this, the ICF was presented and explained, and after the resident signed it, researchers conducted individual interviews using the questionnaire to those who met the inclusion criteria.

The data collection instrument consisted of 30 questions, divided into two parts. A pilot test of the questionnaire was conducted in the communities to adapt it to the population and research objectives. The first part, called socioeconomic characterization, contains questions designed to identify this population in terms of sociodemographic indicators. The second part (health behavior and adherence to the COVID-19 vaccine) was based on the WHO tool “Behavioral and social drivers of vaccination: Practical tools and guidance for achieving high vaccine acceptance rates”^(30)^, which uses behavioral and social drivers (BSD) (specific beliefs and experiences related to vaccination that are potentially modifiable to increase vaccine uptake) to understand what motivates vaccine acceptance. It groups four domains for assessment: 1. thinking and feeling about vaccines; 2. social processes that encourage or inhibit vaccination; 3. motivation (or hesitation) to seek vaccination; 4. practical issues involved in seeking and receiving vaccines^(31)^.

In this study, questions from item 2.2 of the “BSD tool for COVID-19 vaccination” were used and adapted. Specifically, questions 14 to 25 and 28 to 30 were applied in a questionnaire, using a Likert scale of importance (with variations such as “not at all concerned” to “very concerned”; “absolutely nothing” to “very much”; and “not at all important” to “very important”). To meet the research objectives, questions 13, 25, and 29 were added. Moreover, questions 30 and 31 were included to profile the local population regarding the presence of comorbidities and COVID-19 diagnosis.

### Analysis of results and statistics

Using Excel’s pivot table, the data collected in the two communities where the research took place were digitized and organized. A database was then created, into which all the variables investigated in the socioeconomic questionnaire and the questionnaire on health behavior and adherence to the COVID-19 vaccine were entered. Subsequently, descriptive statistical analysis was performed, determining the mean and the absolute and relative frequencies of the variables that comprised the questionnaire. For the socioeconomic questionnaire, the mean, absolute frequencies, and relative frequencies were determined. For health behavior and adherence to the COVID-19 vaccine, the data were grouped into domains according to the “BSD tool for COVID-19 vaccination”^(31)^, applying absolute and relative values for description.

## RESULTS

A total of 152 participants from both communities took part in this research. The study identified that participants’ mean age was 43 years, with a predominance of females (57%), married (86.56%), and having completed primary education or being below that level of education (49%).

Moreover, 54% of the studied population earned more than one minimum wage, while the remaining 46% earned less than one minimum wage. Masonry construction predominated (58%), but stilt houses were also prevalent (32%). Fishermen and farmers accounted for 41% of occupations, with a significant percentage engaged in other activities (49%). Only 37% had comorbidities, and 31% lived with four other people. Means of transportation included canoes with outboard motors (28%), canoes and other means (33% - buses). The predominant religion was Evangelical (52%), and the travel time to the urban center was one hour for 46% and two hours or more for 37%.

Table 1 shows the percentage of COVID-19 vaccine uptake among community residents, demonstrating that the majority (99%) received at least one dose of the vaccine, as well as the number of doses received. The percentage of participants who received one dose was 7%; those who received two doses accounted for 22%; those who received three doses accounted for 30%; those who received four doses comprised 28%; and those who received up to five doses of the COVID-19 vaccine accounted for 13% of those interviewed.

**Table 1:** Distribution of the percentage of adherence to the COVID-19 vaccine in riverside communities (Samaúma and Tilheiro; N=152), Manaus, Amazonas, Brazil, 2024

Variables	n	%
**Have you received any doses of the COVID-19 vaccine?**		
Yes	150	98.68
No	2	1.32
**Number of doses of the COVID-19 vaccine received**		
1	12	7.89
2	33	21.71
3	44	28.95
4	43	28.29
5	20	13.16
**Do you know where to go to have your COVID-19 vaccine?**		
Yes	150	98.68
No	2	1.32
**How is the access to the COVID-19 vaccine?**		
Not easy at all	8	5.30
Somewhat easy	4	2.65
Very easy	122	80.13
Moderately easy	18	11.92
**What is making it difficult for you to receive the COVID-19 vaccine?**		
It is not difficult	73	48.03
It is easy	42	27.63
The waiting time is too long	29	19.08
I cannot go alone. The vaccination site is far away.	3	1.97
Other	2	1.32
It is not difficult	3	1.97
**Where would you prefer to receive a COVID-19 vaccine?**		
Residence	14	9.15%
Basic Health Unit	138	90.85%

*Source: Damasceno (2024)^(23,31)^.*

Table 2 presents the domain of thinking and feeling about vaccines, describing behavior in relation to adherence and non-adherence to the COVID-19 immunization process.

**Table 2:** Distribution of participants according to access to the COVID-19 vaccine in riverside communities (Samaúma and Tilheiro; N=152), Manaus, Amazonas, Brazil, 2024

Variables	n	%
**What are your concerns about being vaccinated against COVID-19?**		
Not at all worried	77	50.66
Slightly worried	39	25.66
Moderately worried	17	11.18
Very worried	19	12.50
**Do you trust the COVID-19 vaccines?**		
Absolutely nothing	21	13.82
A little	23	15.13
Moderately	51	33.55
A lot	57	37.50
**How important is it to you to be vaccinated against COVID-19?**		
Nothing	11	7.24
Little	13	8.55
Moderately	42	27.63
Very	86	56.58
**What is your concern that the vaccines could cause a reaction?**		
Not at all worried	51	33.55
Slightly worried	30	19.74
Moderately worried	32	21.05
Very worried	39	25.66

*Source: Damasceno (2024)^(23,31)^.*

Table 3 presents the social processes that can encourage or inhibit vaccination, with variables that complement behavior regarding adherence and non-adherence to the COVID-19 immunization process.

**Table 3:** Distribution of participants according to access to the COVID-19 vaccine in riverside communities (Samaúma and Tilheiro; N=152), Manaus, Amazonas, Brazil, 2024

Variables	n	%
**Being vaccinated against COVID-19 will protect other people in your community from the disease**		
Absolutely nothing	15	9.87
A little	23	15.13
Moderately	38	25.00
A lot	76	50.00
**Do you think it is important for your family and friends that you receive a COVID-19 vaccine?**		
Yes	128	84.21
No	9	5.92
I am not sure	15	9.87
**Do you think it is important for your community leaders or religious leaders to receive a COVID-19 vaccine?**		
Yes	110	71.90
No	10	6.54
I am not sure	33	21.57
**Do you think receiving a COVID-19 vaccine will allow you to see your family and friends safely?**		
Yes	119	78.29
No	9	5.92
I am not sure	24	15.79
**Have you seen or heard anything negative about COVID-19 vaccines?**		
Yes	123	80.92
No	29	19.08

*Source: Damasceno (2024)^(23,31)^.*

As for motivation for vaccination, 56.29% (56 participants) expressed no intention to be vaccinated. Following this, 25.66% (39 participants) considered vaccination moderately, and 17.76% (27 participants) showed little intention. As for the clinical profile of the population, the majority (62.50%; 95 participants) did not report having comorbidities. Additionally, 56.29% (85 participants) reported not having been diagnosed with COVID-19.

## DISCUSSION

Sociodemographic profiles can influence both the spread of COVID-19 and vaccination adherence^(32,33)^, even with the technological innovations of vaccines^(34)^. In this context, the reluctance to vaccinate stems from contextual, individual/group, and vaccine-specific factors^(35)^.

In this study, most participants had completed high school, suggesting a good level of education. However, it is important to note that a considerable number of individuals had only completed elementary school or less. Regarding income, most respondents reported earning between one and two minimum wages. However, 46% of the sample received less than the minimum wage. This data was also present in a larger study with 492 riverside dwellers^(36)^, which found low levels of education and income below the minimum wage in 41.7% of its population, in addition to economic activity related to fishing and agriculture (62.2%).

Despite the predominance of masonry houses, the presence of stilt houses or wooden houses, characteristic of the riverside population, was observed. The main economic activities identified were fishing and agriculture, complemented by occupations such as public servant, merchant, and barber. Most households housed four or more residents, and means of transport varied from canoes to more modern options such as motorboats and buses. Data from the health survey in riverside communities by Gama *et al*.^(18)^ support these data, showing the prevalence of fishing and agriculture economic activities.

According to Rodrigues, Vieira and Barros^(33)^, higher Gross Domestic Product *per capita* and the number of educational institutions were correlated with greater adherence to the COVID-19 vaccine, indicating the influence of education and income on vaccination outcomes. In Brazil, the “COVID-19 vaccination strategies” in 2024 included riverside communities in the priority group, in addition to considering sociodemographic characteristics^(37)^.

Considering the vaccines available at the start of the vaccination campaign, this study showed that the majority received at least one dose of the vaccine, and approximately 92.11% received two or more doses. In 2024, with the inclusion of the “XBB monovalent COVID-19 vaccine” in the COVID-19 immunization program for priority groups, such as riverside communities, the vaccination schedule became a single dose of this vaccine^(38)^. It should be noted that, at the beginning of COVID-19 immunization in Brazil, the complete schedule consisted of at least two doses of the available vaccines, whether for emergency or definitive use^(11)^. This change highlights that, although the studied population demonstrated remarkable initial adherence, this scenario may change according to new protocols established by the Ministry of Health.

According to Alvarez-Manzo *et al*.^(39)^, the intention to vaccinate is globally heterogeneous and, regionally, may be associated with sociodemographic factors. People living in rural areas tend to have lower adherence to the vaccination schedule due to their work activities or other occupations. In the United States of America, primary care figures, such as pharmacists and physician assistants, are the drivers of information, given the difficult access in rural areas^(40)^. According to Sun and Monnat^(41)^, the vaccination rate in rural areas of the United States of America was lower compared to urban areas.

In general, vaccines represent an efficient mechanism for health. In the case of COVID-19, one of the dilemmas of this process lies in the unequal access to the vaccine itself, the rural-urban divide, hesitancy, and denial^(13)^. In India, rural areas also faced a major impact from the high spread of the virus, affecting lives and livelihoods^(42)^. A combination of socioeconomic factors has increased infections among rural Latino populations in Florida, who are a large group of vaccine hesitant individuals^(43)^.

This study revealed that most participants know where to receive the vaccine and consider access to it very easy, without major difficulties. It also identified the Basic Health Unit as the preferred vaccination location. However, 19.8% of the population reported difficulty with waiting times, and 11.92% see access as moderately easy. Although most participants consider vaccination very important and trust vaccines, a large portion showed little concern about receiving the immunizations. However, it is crucial to highlight the considerable presence of individuals very concerned about side effects. Furthermore, a significant minority expressed great concern about being vaccinated and had absolutely no trust in vaccines. For França Silva, Aguiar and de Siqueira^(44)^, “immunization strategies need to be strengthened to reach more vulnerable groups”.

According to Faye *et al*.^(45)^, in both rural and urban areas, most people were aware of the vaccine and concerned about the risk of becoming infected with SARS-CoV-2. In the Senegal region, 41% distrusted vaccines as being safe, expressing concern about side effects. Danabal *et al*.^(11)^ state that, to alleviate anxiety regarding adverse vaccine effects, it is necessary to raise awareness about the importance of vaccine support, educate about the benefits of vaccine-mediated immunity, create a culture of trust, with involvement in the transparent dissemination of vaccine-related information in the community, as well as providing supportive environments, access to vaccination, and physical, emotional and informational support during and after vaccination.

In this study, regarding the social processes that can encourage or inhibit vaccination, it was evident that the majority considered it very important that vaccination would protect people in the community against the disease. They believe that, for their family, friends, community, and religious leaders, it is important to receive the vaccine, as well as to be able to see their family more safely, even though most have heard something negative about vaccines. According to Buro *et al*.^(43)^, vaccination adoption was associated with barriers related to fear, misinformation, accessibility, and political issues. Faith, self-care, and community resilience emerged as strategies to improve vaccination rates. Community and institutional barriers were also identified in the study.

Alvarez-Manzo *et al*.^(39)^ show that Latin America was profoundly affected by the COVID-19 pandemic, and Brazil was among the countries with a large number of deaths, which highlights the importance of assessing vaccine uptake in this region of the continent. The author discusses that economic barriers, supply chain problems, and attitudes are factors that reflect on the vaccination process. Purnell *et al*.^(40)^ showed a high rate of vaccine acceptance, driven by the desire to protect oneself, family, or community against COVID-19. For them, in vulnerable populations, empathy and information are important, addressing individualities and cultural aspects.

In relation to their motivation to receive the recommended vaccines, more than half of participants expressed having absolutely no intention of wanting to be vaccinated against COVID-19. Hubach *et al*.^(46)^ identified factors that can affect vaccine acceptance, such as a lack of scientifically accurate information about COVID-19 to disseminate in their communities, as well as adverse reactions, side effects, availability, and the need to travel long distances, which also limit the intention to be vaccinated. Lack of information and misinformation can influence traditional groups, making it essential to combat this process^(47)^.

For Roy, Huda and Azam^(48)^, social, cultural, spiritual, psychological and emotional factors are predictors of acceptance or reluctance to take a vaccine. In this study, 7.24% of respondents believe they do not need to be vaccinated. For Ramesh and Sowmyashree^(49)^, the reasons for hesitancy were largely side effects, and not taking it unless infected.

In this study, more than half did not present any comorbidity, which is a decisive factor for complications related to COVID-19, and 56% of the surveyed population had not been diagnosed with COVID-19. This evidence is positive for the population of that locality, considering that chronic diseases increase the chances of negative prognoses in COVID-19 evolution^(50)^, as well as in the face of the spread of COVID-19 to inland areas, representing a threat to the Brazilian Amazon’s riverside populations^(51)^.

### Study limitations

This study faced limitations inherent to the small sample size. The complexity and particularities of traditional riverside communities demand a broader population scope to ensure the representativeness of results. However, regional factors, such as geographic aspects, distance, means of transport, and climatic periods, decisively impacted data collection in the field. Additionally, a scarcity of research addressing topics relevant to these populations was observed. Data regarding confirmed COVID-19 diagnoses, the presence of comorbidities, and vaccine administration were self-reported, without documentary evidence. This is due to the convenience sampling approach, which frequently found participants lacking documentation.

### Contributions to nursing

Therefore, it is hoped that this data will generate reflection, in order to understand that, to strengthen vaccination in riverside communities, it is crucial that nursing invests in health education, combating misinformation and using new access methods for flexible campaigns. Public policies should focus on adapted communication and integrated socioeconomic programs that improve adherence, guaranteeing funding and constant updates for health teams. It is essential to understand the population’s fears and build trust, transforming the positive sentiment about the vaccine.

## CONCLUSIONS

The findings of this study offer an overview of vaccination behavior and adherence in the riverside communities investigated, and it is essential to recognize the particularities of the context analyzed here. The data collected were very close to the sociodemographic characteristics of traditional riverside communities described in the literature, such as monthly income below the minimum wage in most cases. However, changes in access to the communities were observed due to new means of transport and new land routes connecting them to urban centers, thus influencing schooling, housing, transportation, and economic activities. The majority of the surveyed population had completed the two-dose vaccination schedule against COVID-19, according to the 2022 guidelines. However, with the publication of new guidelines from the Ministry of Health in 2024, this scenario is likely to change, considering the peculiarities found in the study, such as trust, fear of vaccines, and the desire to no longer be vaccinated.

It is also noted that, in COVID-19 vaccination, information about vaccines and social factors can contribute negatively or positively. In these communities, a trend of progressive refusal of vaccine doses was observed among respondents, which may influence the single-dose schedule. Vaccine access, location, and availability were not considered problems, nor were difficulties in obtaining them reported. Sentiment regarding the vaccine was positive in terms of confidence, importance, and concern, as well as regarding the social processes that inhibit or encourage vaccination, even though many had received negative information about them.

Considering the study’s limitations, it can be understood that, in the COVID-19 vaccination process in the riverside communities studied, there was a positive relationship between social factors and feelings about COVID-19 vaccination. This occurred even though the communities showed regression in the vaccination process. Despite the advances related to the unique characteristics of this population, there is a lack of information about the importance of vaccines. Finally, it is suggested that future studies could be more comprehensive in communities in order to generate greater data accuracy.

## Data Availability

The research data are available within the article.

## References

[1] V’kovski P, Kratzel A, Steiner S, Stalder H, Thiel V. (2021). Coronavirus biology and replication: implications for SARS-CoV-2. Nat Rev Microbiol.

[2] Tabatabaeizadeh SA. (2021). Airborne transmission of COVID-19 and the role of face mask to prevent it: a systematic review and meta-analysis. Eur J Med Res.

[3] World Health Organization (WHO) (2021). Obtendo a vacina COVID-19.

[4] World Health Organization (WHO) (2021). Segurança das vacinas COVID-19.

[5] Gonçalves BA, Matos CCSA, Ferreira JVS, Itagyba RF, Moço VR, Couto MT. (2023). Hesitação vacinal contra a COVID-19 na América Latina e África: uma revisão de escopo. Cad Saúde Pública.

[6] World Health Organization (WHO) (2021). Acesso e alocação: como haverá alocação justa e equitativa de suprimentos limitados?.

[7] World Health Organization (WHO) (2024). Kit de ferramentas para introdução da vacina COVID-19.

[8] Instituto Butantan (2024). Retrospectiva 2021: segundo ano da pandemia é marcado pelo avanço da vacinação contra Covid-19 no Brasil.

[9] Ministério da Saúde (BR) (2022). Plano nacional de operacionalização da vacinação contra a Covid-19.

[10] Ministério da Saúde (BR) (2023). Atualizações referentes aos esquemas primários e dose de reforço de vacinas COVID-19 em crianças imunocomprometidas de 5 a 11 anos de idade.

[11] Ministério da Saúde (BR) (2023). Inclusão de comorbidades como grupo prioritário para recebimento de dose de reforço com a vacina COVID-19 bivalente.

[12] Ministério da Saúde (BR) (2024). Informe técnico: inclusão da vacina COVID-19 monovalente XBB na estratégia de vacinação contra a COVID-19.

[13] Danabal KGM, Magesh SS, Saravanan S, Gopichandran V. (2021). Attitude towards COVID 19 vaccines and vaccine hesitancy in urban and rural communities in Tamil Nadu, India: a community based survey. BMC Health Serv Res.

[14] Purnell M, Maxwell T, Hill S, Patel R, Trower J, Wangui L (2022). Exploring COVID-19 vaccine hesitancy at a rural historically black college and university. J Am Pharm Assoc.

[15] Bonfá D, Domingos BS, Silva IR. (2021). Direitos dos ribeirinhos no Brasil: construção de barragens e a pandemia COVID-19. Rev Verde Grande.

[16] Presidência da República (BR) (2007). Institui a Política Nacional de Desenvolvimento Sustentável dos Povos e Comunidades Tradicionais.

[17] Ministério do Meio Ambiente e Mudança do Clima (BR) (2025). Povos e Comunidades Tradicionais.

[18] Gama ASM, Fernandes TG, Parente RCP, Secoli SR. (2018). Inquérito de saúde em comunidades ribeirinhas do Amazonas, Brasil. Cad Saude Publica.

[19] Michelon CM. (2021). Principais variantes do SARS-CoV-2 notificadas no Brasil. RBAC.

[20] Ministério da Saúde (BR) (2025). Painel de casos de doença pelo coronavírus 2019 (COVID-19).

[21] Secretaria de Saúde do Estado do Amazonas, Fundação de Vigilância em Saúde (2025). Painel Epidemiológico COVID-19 Amazonas.

[22] Secretaria de Saúde do Estado do Amazonas, Fundação de Vigilância em Saúde (2025). Vacinômetro COVID-19 Amazonas.

[23] Damasceno HA. (2024). Vacinas covid-19: adesão ao esquema vacinal em comunidades ribeirinhas do município do Careiro/AM.

[24] Rozin L. (2020). Em tempos de COVID-19: um olhar para os estudos epidemiológicos observacionais. Rev Espaço Saúde.

[25] Luz ALA, Oliveira EAR, Torres CRD, De Carvalho KM, Monteiro CFS, Moura MEB. (2015). Abordagens quantitativa e qualitativa nas pesquisas em saúde. Rev Enferm UFPI.

[26] Instituto de Desenvolvimento Agropecuário e Florestal Sustentável do Estado do Amazonas (IDAM) (2012). Careiro.

[27] Instituto Brasileiro de Geografia e Estatística (IBGE) (2022). Careiro: panorama.

[28] Castro V (2019). Urbanização contemporânea na Amazônia e os fragmentos de cidade: o caso do Município de Careiro e as vilas Araçá, Purupuru e Samaúma.

[29] Costa SD, Leite MJD, Farias JPB. (2019). In: Anais do Fórum HABITAR 2019: Habitação e Desenvolvimento Sustentável; 2019; Belo Horizonte.

[30] Oliveira MOR, Luce FB, Sampaio CH, Perin MG, Santini FO, Santos J. (2017). Análise da qualidade dos artigos científicos da área de marketing publicados no Brasil: as pesquisas survey na década de 2000. REAd Rev Eletrôn Adm (Porto Alegre).

[31] Organização Mundial da Saúde (OMS) (2022). Motores comportamentais e sociais da vacinação: ferramentas e orientações práticas para se atingir uma elevada taxa de aceitação das vacinas.

[32] Arnaldo C, Arau A, Hansine R, Niquisse S, Jasper P, Matsinhe C (2020). Características sociodemográficas e risco de transmissão da COVID-19 em Moçambique. Rev Moçamb Ciênc Saúde.

[33] Rodrigues BF, Vieira MF, Barros GBS. (2024). Estudo epidemiológico da correlação entre fatores sociodemográficos e adesão à vacinação contra a COVID-19 na macrorregião Sul de Minas Gerais. Res Soc Dev.

[34] Ministério da Saúde (BR) (2024). Manual de normas e procedimentos para vacinação.

[35] Organização Pan-Americana da Saúde (Opas) (2020). Como se comunicar sobre a segurança das vacinas: diretrizes para orientar os trabalhadores da saúde quanto à comunicação com pais, mães, cuidadores e pacientes.

[36] Facundes GA, Maciel BVL, Silva MP, Alves PJK, Silva RMH, Gama SM. (2020). Acesso a serviços de saúde por ribeirinhos de um município no interior do estado do Amazonas, Brasil. Rev Pan Amaz Saude.

[37] Ministério da Saúde (BR) (2024). Plano nacional de operacionalização da vacinação contra a Covid-19.

[38] Ministério da Saúde (BR) (2024). Inclusão da vacina covid-19 monovalente XBB na estratégia de vacinação contra a COVID-19.

[39] Alvarez-Manzo HS, Badillo-Davila R, Olaya-Gomez A, Gonzalez-de-Cossio-Tello B, Cardoso-Arias R, Gamboa-Balzaretti ES (2021). COVID-19 Vaccine Intention among Rural Residents in Mexico: validation of a questionnaire. Vacinas.

[40] Purnell M, Maxwell T, Hill S, Patel R, Trower J, Wangui L (2022). Exploring COVID-19 vaccine hesitancy at a rural historically black college and university. J Am Pharm Assoc.

[41] Sun Y, Monat SM. (2022). Rural-urban and within-rural differences in COVID-19 vaccination rates. J Rural Health.

[42] George CE, Inbaraj LR, Rajukutty S, Joan RF, Suseeladevi AK, Muthuraj S (2022). Seroprevalence of COVID-19 infection among vaccine naïve population after the second surge (June 2020) in a rural district of South India: a community-based cross-sectional study. PLOS ONE.

[43] Buro AW, Roman Candelaria K, Bailey R, Luna F, Albizu-Jacob A, Stern M (2022). Exploration of multilevel barriers and strategies that affected early COVID-19 vaccination and testing in rural Latino communities in Southwest Florida. Int J Environ Res Public Health.

[44] Silva KNF, Aguiar LR, Siqueira MT. (2023). Vacinação contra Covid-19 no distrito sanitário III de Recife - PE, Brasil, em 2021 e 2022. Rev Eletr Acervo Saúde.

[45] Faye SLB, Krumkamp R, Doumbia E, Tounkara M, Strauss R, Ouedraogo HG (2022). Factors influencing hesitancy towards adult and child COVID-19 vaccines in rural and urban West Africa: a cross-sectional study. BMJ Open.

[46] Shannon B, Morgan KD, Alexander C, O’Neil AM, Ernst C, Giano Z. (2022). COVID-19 vaccine hesitancy among rural Oklahomans. Rural Remote Health.

[47] Matos RSC, Costa AMS, Marcelino RS, Gomes VO, Folhadela RE, Souza GKP (2022). Adesão das comunidades ribeirinhas a vacinação contra a Covid-19 no interior do Amazonas. Rev Eletr Acervo Saúde.

[48] Roy DN, Huda MN, Azam MS. (2022). Factors influencing COVID-19 vaccine acceptance and hesitancy among rural community in Bangladesh: a cross-sectional survey based study. Hum Vaccin Immunother.

[49] Ramesh M, Sowmyashree U. (2021). Awareness regarding COVID-19 vaccine in a rural area near Bangalore, Karnataka. Natl J Community Med.

[50] Feitoza TMO, Chaves AM, Muniz GTS, Cruz MCC, Cunha IF. (2020). Comorbidades e COVID-19. Rev Interfaces.

[51] Castro FF, Souza CRS, Diniz CX, Parmejiani EP, Santos FS, Nascimento JN, Santana RF (2020). Idosos ribeirinhos da Amazônia Brasileira no enfrentamento da covid-19. Enfermagem gerontológica no cuidado do idoso em tempos da COVID 19.

