# Supplementary material for: Adherence to COVID-19 vaccination in riverside populations of the municipality of Careiro/AM
Source: Rev Bras Enferm. 2026 Aug 7;79:e20250066. doi: 10.1590/0034-7167-2025-0066 (PMC13450938; doi:10.1590/0034-7167-2025-0066)
Supplement: Translation

# Translation: Adesão à vacinação contra a COVID-19 em populações ribeirinhas do município do Careiro/AM

**DOI:** 10.1590/0034-7167-2025-0066pt

**Authors:** Hibelfran Alfaia Damasceno, Vivian Cristine de Souza e Souza, Luiz Henrique Gonçalves Maciel, Marcel Gonçalves Maciel

**Affiliations:** I Universidade Federal do Amazonas. Manaus, Amazonas, Brasil; II Universidade do Estado do Amazonas. Manaus, Amazonas, Brasil

**Keywords:** Vacinação, Esquemas de Imunização, COVID-19, População Rural, População Ribeirinha.

## Abstract

**Objetivos:**

descrever a adesão ao esquema vacinal da COVID-19 em duas comunidades ribeirinhas do Careiro/AM.

**Métodos:**

estudo epidemiológico, observacional, descritivo e transversal, com abordagem quantitativa, realizado em 2023-2024. Dados foram coletados via entrevista, guiados por formulário padronizado, analisados descritivamente no *Excel* (valores relativos e absolutos).

**Resultados:**

características sociodemográficas foram similares à literatura. A maioria recebeu ao menos uma dose (98,68%). Acesso, preocupação e fatores sociais influenciaram a vacinação. A não vontade de se vacinar predominou (36,84%). Foram identificados 62,50% sem comorbidades e 56,29% com diagnostico diagnóstico informado de COVID-19.

**Conclusões:**

as comunidades ribeirinhas pesquisadas estão passando por transformações. Além disso, elas exibem uma recusa progressiva das doses da vacina, que parecem estar associadas a fatores sociais e à informação sobre as vacinas.

## INTRODUÇÃO

O SARS-CoV-2 (Coronavírus da Síndrome Respiratória Aguda Grave 2), responsável pela pandemia de COVID-19, causa doença infecciosa respiratória e afeta qualquer pessoa, sendo capaz de manifestar formas leves ou graves^(1)^. A transmissão, que ocorre por via respiratória, através de gotículas ou aerossóis, está relacionada à alta transmissibilidade^(2)^. Nesse cenário, diversas iniciativas mundiais foram desenvolvidas e aplicadas visando conter a pandemia.

As vacinas, uma dessas iniciativas alavancadas em 2020, representam ferramentais indispensáveis na luta contra a COVID-19, pois garantem prevenção individual e coletiva de formas graves da doença, por meio da sensibilização do sistema imunológico do corpo para reconhecer e criar anticorpos contra o vírus. No entanto, podem causar efeitos colaterais leves ou graves, que tendem a desaparecer em alguns dias^(3,4)^. A imunização é indispensável no controle da propagação da pandemia de COVID-19^(5)^.

A Organização Mundial da Saúde liderou o Centro de Acesso Global de Vacinas COVID-19 (iniciativa *COVAX Facility*), que otimizou o cronograma das produções e licenciamento das vacinas^(6)^. Protagonizou, ainda, a construção de um *kit* de ferramentas, com orientações, treinamento e informações para apoiar programas de distribuição das vacinas. Paralelamente a esses esforços, foram conduzidos estudos controlados randomizados, a fim de atestar a segurança e eficácia das vacinas^(7)^.

No Brasil, apesar de o início da vacinação ter ocorrido em 2021, utilizando vacinas com autorização de uso emergencial, foi em 2022 que houve o lançamento do primeiro Plano Nacional de Operacionalização da Vacinação contra COVID-19^(8,9)^, sendo atualizado em 2023^(10,11)^. A partir de junho de 2024, foi introduzida no Brasil uma nova diretriz com esquema vacinal utilizando a vacina monovalente XBB, fabricada pela empresa Moderna. As orientações consideravam diferentes faixas etárias e grupos prioritários, como as comunidades ribeirinhas^(12)^.

Contudo, desafios persistem, como a hesitação vacinal, manifestando-se na resistência de indivíduos em aceitar as vacinas, mesmo sendo seguras, eficazes e acessíveis para prevenção de doenças^(13,14)^. Para as comunidades ribeirinhas, que possuem determinantes sociais específicos, as ações de vacinação devem considerar os desafios econômicos e logísticos, visto que, em áreas mais remotas, vacinações escalonadas não apresentam um custo-benefício positivo, correndo o risco de introdução do vírus^(11)^.

Os povos ribeirinhos estão localizados às margens de “igarapés, igapós, lagos e várzeas”^(15)^, e são classificados como parte dos povos tradicionais não indígenas, que surgiram da miscigenação colonial e migratória^(16,17)^. A associação entre as desfavoráveis condições econômicas, mudanças climáticas e limitação geográfica representa barreiras para o acesso à saúde nessas comunidades. Além disso, os ribeirinhos possuem um estilo de vida singular, com comportamentos laborais e sociais coletivos fortemente baseados em sua estrutura familiar e comunitária, cujo acesso requer logística especializada^(18)^.

A COVID-19 é uma doença que apresenta alta transmissíbilidade que, em pouco tempo, já apresentou mutações gênicas do vírus SARS-CoV-2^(19)^. No Brasil, houve alta disseminação da doença, evidenciada no número de casos novos. Até 2024, na região Norte, foram registrados acima de 2 milhões de casos confirmados e mais de 52 mil óbitos^(20)^. O estado do Amazonas, por sua vez, acumulou 643.730 casos e 14.528 óbitos. A capital, Manaus, com mais de 2 milhões de habitantes, concentrou mais de 190 mil casos e 9 mil óbitos. Já o município do Careiro, na Região Metropolitana de Manaus, com população de 37.869 habitantes (Instituto Brasileiro de Geografia e Estatística, 2022), contabilizou 6.900 casos confirmados e 93 óbitos^(21)^.

Diante desse cenário, as vacinas contra a COVID-19 representaram uma ferramenta indispensável na proteção e controle da pandemia. No entanto, no município do Careiro, que possui uma significativa população rural, incluindo comunidades ribeirinhas, os registros estaduais indicam uma queda na cobertura vacinal entre a primeira e a segunda dose, atingindo 69,4% e 58,5%, respectivamente. Os índices de reforço apresentaram uma redução ainda mais acentuada, com o primeiro reforço alcançando 34,5%, e o segundo, 12,9%. Adicionalmente, o esquema primário de vacinação atingiu apenas 61% da população total^(21,22)^. Considerando o exposto sobre a pandemia de COVID-19 e as características específicas das comunidades tradicionais ribeirinhas, o presente projeto propôs responder: como ocorre a adesão ao esquema vacinal da COVID-19 em duas comunidades tradicionais ribeirinhas do município do Careiro/AM?

## Relevância do estudo

O presente manuscrito trata-se de recorte do estudo metodológico oriundo da dissertação^(23)^ intitulada “Vacinas covid-19: adesão ao esquema vacinal em comunidades ribeirinhas do município do Careiro/AM”.

## OBJETIVOS

Descrever a adesão ao esquema vacinal das vacinas COVID-19 em duas comunidades ribeirinhas do município do Careiro/AM.

## MÉTODOS

### Aspectos éticos

O estudo foi guiado obedecendo as diretrizes de éticas nacionais e internacionais, de acordo com a Resolução nº 466/12, sendo aprovado pelo Comitê de Ética em Pesquisa (CEP) do Universidade Federal do Amazonas, conforme Certificado de Apresentação de Apreciação Ética n^o^ 71215323.4.0000.5020. A coleta teve início após o projeto ser aprovado no referido CEP. Para todos os envolvidos, foi aplicado o Termo de Consentimento Livre e Esclarecido (TCLE) escrito.

### Desenho, período e local do estudo

Está traçado dentro de estudo epidemiológico, observacional, descritivo e transversal, com abordagem quantitativa^(24,25)^. A pesquisa seguiu o *STrengthening the Reporting of OBservational studies in Epidemiology* da rede EQUATOR. A pesquisa foi realizada em uma das 39 comunidades^(26)^, pertencentes ao município do Careiro (Tilheiro e Samaúma), realizadas em meses de 2023 e 2024, por meio de visitas presenciais. Careiro é um município do Amazonas que pertence à Região Metropolitana de Manaus. De acordo com os indicadores municipais, a maioria da população reside na zona rural (23.297 pessoas)^(27)^. As comunidades selecionadas para compor a área e a população do estudo são Tilheiro e Sumaúma, que possuem as características típicas de comunidades tradicionais ribeirinhas^(28,29)^.

### População e amostra

A abordagem ocorreu, de maneira sequencial, durante as visitas domiciliares aos participantes da pesquisa, selecionando um a cada cinco domicílios. Diante do aspecto peculiar das comunidades pesquisadas, que incluem o difícil acesso devido à localização geográfica e às condições de transporte, facilitadas pelos ciclos de sazonais típicos da Amazônia, foi aplicada amostra não probabilística por conveniência, na qual os elementos da pesquisa são selecionados de acordo com a conveniência ou facilidade para o pesquisador^(30)^.

### Critérios de inclusão e exclusão

Incluíram-se pessoas com idade igual ou acima de 18 anos, residindo na área de estudo, capazes de compreender as questões da entrevista (avaliada pela percepção do entrevistador). Excluíram-se pessoas com contraindicações específicas para vacina contra a COVID-19 (hipersensibilidade aos princípios ativos da vacina), deficiência mental (informada pelo responsável presente), dificuldade de comunicação (avaliada pela percepção do entrevistador), sem condições de saúde favoráveis no momento da visita (sintomas agudos ou graves, comprometimento do estado de consciência/cognição, avaliada pela percepção do entrevistador), idade inferior a 18 anos e que não são capazes de compreender as questões da entrevista (avaliada pela percepção do entrevistador).

### Protocolo do estudo

A coleta de dados foi conduzida por meio de entrevista guiada por formulário padronizado, para obtenção de informações socioeconômicas, clínicas e de comportamento em relação à vacinação contra COVID-19. Foi realizada visita presencial nas comunidades do Tilheiro e Samaúma. A abordagem ocorreu nas residências dos moradores de forma aleatória, sendo explicados os objetivos da pesquisa. Em seguida, foi apresentado e explicado o TCLE, e após assinatura pelo morador, foi aplicada entrevista guiada por formulário pelos pesquisadores, de forma individual, aos que se encaixaram nos critérios de inclusão.

Instrumento da coleta de dados foi composto de 30 questões, divididas em duas partes. Para aplicação nas comunidades, foi realizado teste piloto com o formulário, a fim de adaptá-lo conforme a população e os objetivos da pesquisa. A primeira, chamada de caracterização socioeconômica, contém perguntas que buscam identificar essa população quanto aos indicadores sociodemográficos. A segunda parte (comportamento de saúde e adesão à vacina contra a COVID-19) foi embasada na ferramenta da OMS “Motores comportamentais e sociais da vacinação: Ferramentas e orientações práticas para se atingir uma elevada taxa de aceitação das vacinas”^(31)^, que utiliza os motores comportamentais e sociais (MCS) (convicções e experiências específicas para vacinação que são potencialmente modificáveis para aumentar a captação de vacinas) para compreender o que motiva a aceitação das vacinas. Ela agrupa quatro domínios para avaliação: 1. pensar e sentir acerca das vacinas; 2. processos sociais que incentivam ou inibem a vacinação; 3. motivação (ou hesitação) em recorrer à vacinação; 4. questões práticas envolvidas na procura e toma das vacinas^(31)^.

Neste estudo, foram utilizadas e adaptadas questões do item 2.2 da “Ferramenta MCS para a vacinação contra a COVID-19”. Especificamente, as questões 14 a 25 e 28 a 30 foram aplicadas em um questionário, utilizando uma escala Likert de importância (com variações como “nada preocupado” a “muito preocupado”; “absolutamente nada” a “muito”; e “nada importante” a “muito importante”). Para atender aos objetivos da pesquisa, as questões 13, 25 e 29 foram adicionadas. Além disso, as questões 30 e 31 foram incluídas para traçar o perfil da população local quanto à presença de comorbidades e diagnóstico de COVID-19.

### Análise dos resultados e estatística

Com auxílio da tabela dinâmica do *Excel*, foram realizadas digitalização e organização dos dados coletados nas duas comunidades onde ocorreu a pesquisa. A partir de então, foi criado um banco de dados, no qual foram inseridas todas as variáveis investigadas no questionário socioeconômico e no questionário sobre comportamento de saúde e adesão à vacina contra a COVID-19. Posteriormente, foi feita a análise estatística descritiva, determinando a média e as frequências absolutas e relativas das variáveis que compuseram o questionário. Para o questionário socioeconômico, foram determinadas a média, as frequências absolutas e frequências relativas. Para o comportamento de saúde e adesão à vacina contra a COVID-19, foram agrupados em domínios de acordo com a “Ferramenta MCS para a vacinação contra a COVID-19”^(31)^, aplicando-se os valores absolutos e relativos para descrição.

## RESULTADOS

Participaram desta pesquisa um total de 152 participantes nas duas comunidades. O estudo identificou que a média de idade dos participantes era de 43 anos, predominando o sexo feminino (57%), com estado civil de casado (86,56%), possuiu ensino fundamental completo ou estando abaixo desse nível de instrução (49%).

A renda de 54% da população estudada estava acima de um salário mínimo, e o restante (46%) tinha menos de um salário. Na moradia, predominou (58%) o estilo alvenaria, mas com grande expressividade de palafitas também (32%). A ocupação de pescador e agricultor foi de 41%, com um grande percentual de outras atividades (49%). Somente 37% possuíam alguma comorbidade, e 31% residiam com quatro pessoas. Os meios de transporte utilizados incluíram a utilização da canoa com motor de polpa (28%), canoa e outros (33% - ônibus). A religião predominante foi evangélica (52%), e o tempo de chegada no centro urbano foi de uma hora, para 46%, e de duas horas ou mais, para 37%.

Através da Tabela 1, é possível identificar o percentual de adesão à vacina contra a COVID-19 pelos moradores das comunidades, evidenciando que a maioria (99%) recebeu alguma dose da vacina, além de mostrar o quantitativo de doses recebidas da vacina. O percentual de participantes que recebeu uma dose foi de 7%; aqueles que receberam duas doses representaram 22%; os que receberam três doses representaram 30%; os que receberam quatro doses concentraram 28%; e os que receberam até cinco doses da vacina contra a COVID-19 representaram 13% dos entrevistados.

**Tabela 1:** Distribuição do percentual de adesão à vacina contra a COVID-19 em comunidades ribeirinhas (Samaúma e Tilheiro; N=152), Manaus, Amazonas, Brasil, 2024

Variáveis	n	%
**Recebeu alguma dose da vacina contra a COVID-19?**		
Sim	150	98,68
Não	2	1,32
**Quantidade de doses recebidas da vacina contra a COVID-19**		
1	12	7,89
2	33	21,71
3	44	28,95
4	43	28,29
5	20	13,16
**Sabe onde se dirigir para receber a sua vacina contra a COVID-19?**		
Sim	150	98,68
Não	2	1,32
**Como é o acesso para conseguir a vacina contra a COVID-19?**		
Nada fácil	8	5,30
Um pouco fácil	4	2,65
Muito fácil	122	80,13
Moderadamente fácil	18	11,92
**O que lhe dificulta obter a vacina contra a COVID-19?**		
Não é difícil	73	48,03
É fácil	42	27,63
O tempo de espera é demasiado longo	29	19,08
Não posso ir sozinho	3	1,97
Local de vacinação é longe	2	1,32
Outro	3	1,97
**Onde prefere receber uma vacina contra a COVID-19?**		
Residência	14	9,15%
Unidade Básica de Saúde	138	90,85%

Fonte: (23,31).

A Tabela 2 apresenta o domínio pensar e sentir acerca das vacinas, descrevendo o comportamento em relação à adesão e não adesão ao processo de imunização contra a COVID-19.

**Tabela 2:** Distribuição dos participantes segundo o acesso à vacina contra a COVID-19 em comunidades ribeirinhas (Samaúma e Tilheiro; N=152), Manaus, Amazonas, Brasil, 2024

Variáveis	n	%
**Qual a sua preocupação em tomar as vacinas contra a COVID-19?**		
Nada preocupado	77	50,66
Pouco preocupado	39	25,66
Moderadamente preocupado	17	11,18
Muito preocupado	19	12,50
**Confia nas vacinas contra a COVID-19?**		
Absolutamente nada	21	13,82
Um pouco	23	15,13
moderadamente	51	33,55
Muito	57	37,50
**Qual a importância para você obter as vacinas contra a COVID-19?**		
Nada	11	7,24
Pouco	13	8,55
Moderadamente	42	27,63
Muito	86	56,58
**Qual sua preocupação que as vacinas poderiam causar uma reação?**		
Nada preocupado	51	33,55
Pouco preocupado	30	19,74
Moderadamente preocupado	32	21,05
Muito preocupado	39	25,66

Fonte: (23,31).

A Tabela 3 apresenta os processos sociais que podem incentivar ou inibir a vacinação, com variáveis que complementam o comportamento em relação à adesão e não adesão ao processo de imunização contra a COVID-19.

**Tabela 3:** Distribuição dos participantes segundo o domínio de acesso à vacina contra a COVID-19 em comunidades ribeirinhas (Samaúma e Tilheiro; N=152), Manaus, Amazonas, Brasil, 2024

Variáveis	n	%
**Vacinar-se contra a COVID-19 irá proteger outras pessoas na sua comunidade contra a doença**		
Absolutamente nada	15	9,87
Um pouco	23	15,13
moderadamente	38	25,00
Muito	76	50,00
**Pensa que para sua família e amigos é importante que você receba uma vacina contra a COVID-19?**		
Sim	128	84,21
Não	9	5,92
Não tenho certeza	15	9,87
**Pensa que, para seus dirigentes comunitários ou líderes religiosos, é importante que receba uma vacina contra a COVID-19?**		
Sim	110	71,90
Não	10	6,54
Não tenho certeza	33	21,57
**Pensa que receber uma vacina contra a COVID-19 lhe permiti ver a sua família e amigos com segurança?**		
Sim	119	78,29
Não	9	5,92
Não tenho certeza	24	15,79
**Viu ou ouviu algo negativo sobre as vacinas contra a COVID-19?**		
Sim	123	80,92
Não	29	19,08

Fonte: (23,31).

No que tange à motivação para vacinação, 56,29% (56 participantes) expressaram nenhuma intenção de se vacinar. Em seguida, 25,66% (39 participantes) consideraram a vacinação moderadamente, e 17,76% (27 participantes) demonstraram pouca intenção. Quanto ao perfil clínico da população, a maioria (62,50%; 95 participantes) não relatou possuir comorbidades. Adicionalmente, 56,29% (85 participantes) informaram não ter sido diagnosticados com COVID-19.

## DISCUSSÃO

O perfil sociodemográfico pode influenciar tanto a propagação da COVID-19 quanto a adesão à vacinação^(32,33)^, mesmo com as inovações tecnológicas das vacinas^(34)^. Nesse contexto, a presença da relutância em se vacinar decorre de fatores contextuais, individuais/de grupo e específicos da própria vacina^(35)^.

Neste estudo, a maioria dos participantes apresentava escolaridade até o ensino médio completo, o que sugere um bom nível de instrução. No entanto, é importante notar que um número considerável de indivíduos possuía apenas o ensino fundamental ou menos. Em relação à renda, a maior parte dos entrevistados declarou receber entre um e dois salários mínimos. Contudo, 46% da amostra recebia menos de um salário mínimo. Esses dados estiveram presentes em estudo maior com 492 ribeirinhos^(36)^, que encontrou baixa escolaridade e renda inferior a um salário mínimo em 41,7% da sua população, além de atividade econômica relacionada à pesca e agricultura (62,2%).

Apesar da predominância de residências de alvenaria, observou-se a presença de casas de palafitas ou madeira, característica da população ribeirinha. As principais atividades econômicas identificadas foram pesca e agricultura, complementadas por ocupações como servidor público, comerciante e barbeiro. A maioria dos lares abrigava quatro ou mais residentes, e os meios de transporte variavam desde canoas até opções mais modernas como lanchas motorizadas e ônibus. Os dados do inquérito de saúde em comunidades ribeirinhas de Gama *et al.* ^(18)^ corroboram esses dados, mostrando a prevalência das atividades econômicas de pesca e agricultura.

De acordo com Rodrigues, Vieira e Barros^(33)^, o maior Produto Interno Bruto *per capita* e o número de instituições de ensino foram correlacionados com maior adesão à vacina contra a COVID-19, indicando a influência da escolaridade e da renda nos resultados da vacinação. No Brasil, as “estratégias de vacinação contra a COVID-19”, em 2024, incluíram as comunidades ribeirinhas no grupo prioritário, além de considerar as características sociodemográficas^(37)^.

Considerando as vacinas disponíveis no início da vacinação, neste estudo, foi evidenciado que a maioria recebeu no mínimo uma dose da vacina e cerca de 92,11% receberam duas ou mais doses do imunizante. Em 2024, com a inserção da “vacina COVID-19 monovalente XBB” no programa de imunização contra a COVID-19, para os grupos prioritários, como os ribeirinhos, o esquema vacinal passou a ser uma dose desse imunizante^(38)^. Salienta-se que, no início da imunização contra a COVID-19 no Brasil, o esquema completo era de pelo menos duas doses dos imunizantes disponíveis, seja de uso emergencial ou definitivo^(11)^. Essa mudança destaca que, embora a população estudada tenha demonstrado uma adesão inicial notável, esse panorama pode ser alterado de acordo os novos protocolos firmados pelo Ministério da Saúde.

Para Alvarez-Manzo *et al*.^(39)^, intenção de se vacinar é mundialmente heterogênea e, regionalmente, pode estar associada a fatores sociodemográficos. Pessoas que vivem em áreas rurais tendem a ter menor adesão ao esquema vacinal, devido ao exercício de suas atividades laborais ou outras ocupações. Nos Estados Unidos da América, as figuras da atenção primária, como farmacêuticos e assistentes médicos, são os condutores de informação, diante do acesso dificultado em áreas rurais^(40)^. De acordo com Sun e Monnat^(41)^, a taxa de vacinação nas áreas rurais dos Estados Unidos da América era menor em relação às áreas urbanas.

De modo geral, as vacinas representam um mecanismo eficiente para a saúde. No caso da COVID-19, um dos dilemas desse processo se apresenta no próprio acesso desigual à vacina, na divisão rural-urbana, na hesitação e na negação^(13)^. Na Índia, as áreas rurais também enfrentaram grande impacto com a alta disseminação do vírus, afetando a vida e os meios de subsistência^(42)^. Um conjunto de fatores socioeconômicos aumentou as infecções entre populações rurais latinas na Flórida, sendo estas grandes representantes dos hesitantes vacinais^(43)^.

Este estudo revelou que a maioria dos participantes sabe onde obter a vacina e considera o acesso a ela muito fácil, sem grandes dificuldades para obtenção. Também identificou que a Unidade Básica de Saúde é o local de vacinação preferido. No entanto, 19,8% da população relatou dificuldade com o tempo de espera, e 11,92% veem o acesso como moderadamente fácil. Apesar de a maioria dos participantes considerar a vacinação muito importante e confiar nas vacinas, grande parte se mostrou pouco preocupada em tomar os imunizantes. É crucial destacar, contudo, a presença considerável de indivíduos muito preocupados com as reações. Além disso, uma minoria significativa expressou muita preocupação em se vacinar e não confiava absolutamente nas vacinas. Para França Silva, Aguiar e de Siqueira^(44)^, “estratégias de imunização precisam ser fortalecidas para alcançar grupos mais vulnerabilizados”.

Para Faye *et al*.^(45)^, tanto em áreas rurais quanto em áreas urbanas, a maioria das pessoas estava ciente sobre a vacina e preocupada com o risco de se infectar pelo SARS-CoV-2. Na região do Senegal, 41% desacreditavam nas vacinas como sendo seguras, manifestando preocupação com os efeitos colaterais. Danabal *et al.* ^(11)^ dizem que, para aliviar a ansiedade em relação aos efeitos adversos da vacina, é necessário sensibilizar sobre a importância do suporte à vacinação, conscientizar sobre os benefícios da imunidade mediada por vacinas, criar uma cultura de confiança, com o envolvimento na disseminação transparente de informações relacionadas à vacina na comunidade, além de proporcionar ambientes favoráveis, acesso à vacinação, e suporte físico, emocional e informativo durante e após a vacinação.

Neste estudo, no item sobre os processos sociais que podem incentivar ou inibir a vacinação, evidenciou-se que a maioria considerava muito importante que a vacinação protegeria as pessoas da comunidade contra a doença. Acreditam que, para sua família, amigos, líderes comunitários e religiosos, é importante receber a vacina, assim como poder ver sua família com mais segurança, mesmo que a maioria tenha ouvido algo negativo sobre as vacinas. De acordo com Buro *et al*.^(43)^, a adoção da vacinação foi associada a barreiras relacionadas ao medo, à desinformação, à acessibilidade e às questões políticas. A fé, o cuidado consigo mesmo e a resiliência da comunidade surgiram como estratégias para melhorar as taxas de vacinação. Barreiras comunitárias e institucionais também foram identificadas no estudo.

Alvarez-Manzo *et al*.^(39)^ mostram que a América Latina foi profundamente afetada pela pandemia de COVID-19, e o Brasil estava entre os países com grande número de óbitos, o que destaca a importância de avaliar a adesão vacinal nessa região do continente. O autor discute que barreiras econômicas, problemas na cadeia de suprimentos e atitudes são fatores que refletem no processo de vacinação. Purnell *et al*.^(40)^ mostraram uma alta taxa de aceitação da vacina, impulsionada pela vontade de proteger a si, a família ou a comunidade contra a COVID-19. Para eles, em populações vulneráveis, a empatia e a informação são importantes, atendendo às individualidades e à culturalidade.

Sobre a motivação em tomar as vacinas recomendadas, mais da metade dos participantes manifestou não ter absolutamente nenhuma intenção em querer tomar as vacinas contra a COVID-19. Hubach *et al*.^(46)^ identificaram fatores que podem afetar a aceitação da vacina, como a falta de informação cientificamente precisa sobre a COVID-19 para propagar em suas comunidades, bem como reações adversas, efeitos colaterais, disponibilidade e a necessidade de percorrer longas distâncias, que também limitam a intenção de se vacinar. A falta de informação e notícias inverídicas pode influenciar grupos tradicionais, sendo fundamental combater esse processo^(47)^.

Para Roy, Huda e Azam^(48)^, fatores sociais, culturais, espirituais, psicológicos e emocionais são preditores da aceitação ou recua de uma vacina. Neste estudo, 7,24% dos entrevistados acreditam que não precisam ser vacinados. Para Ramesh e Sowmyashree^(49)^, os motivos da hesitação foram os efeitos colaterais, em grande parte, e não tomar a menos que for infectado.

Neste estudo, mais da metade não apresentava nenhuma comorbidade, que é um fator decisivo para complicações relacionadas à COVID-19, e 56% da população pesquisada não havia sido diagnosticada com COVID-19. Essas evidências são positivas para a população daquela localidade, considerando que as doenças crônicas aumentam as chances de prognósticos negativos na evolução da COVID-19^(50)^, bem como diante da disseminação da COVID-19 para áreas do interior, representando uma ameaça para as populações ribeirinhas da Amazônia brasileira^(51)^.

### Limitações do estudo

O presente estudo enfrentou limitações inerentes ao quantitativo de participantes na amostra. A complexidade e as particularidades das comunidades tradicionais ribeirinhas demandam uma abrangência populacional maior para assegurar a representatividade dos resultados. Contudo, fatores regionais, como aspectos geográficos, distância, meios de transporte e períodos climáticos, impactaram decisivamente a coleta de dados em campo. Adicionalmente, observou-se uma escassez de pesquisas abordando temas relevantes para essas populações. Dados referentes à confirmação diagnóstica de COVID-19, à presença de comorbidades e à administração de vacinas foram autorreferidos, sem comprovação documental. Tal ocorrência se deve ao critério de abordagem por conveniência, que frequentemente encontrava os participantes em situações desprovidas de suas documentações.

### Contribuições para a área da enfermagem

Desse modo, espera-se que esses dados possam gerar uma reflexão, a fim de compreender que, para fortalecer a vacinação em comunidades ribeirinhas, é crucial que a enfermagem invista em educação em saúde, combatendo a desinformação e utilizando os novos acessos para campanhas flexíveis. As políticas públicas devem focar em comunicação adaptada e em programas socioeconômicos integrados que melhorem a adesão, garantindo financiamento e atualização constante para as equipes de saúde. É fundamental entender os medos da população e construir confiança, transformando o sentimento positivo sobre a vacina.

## CONCLUSÕES

Os achados deste trabalho oferecem um panorama sobre o comportamento e adesão à vacinação nas comunidades ribeirinhas investigadas, sendo fundamental reconhecer as particularidades do contexto aqui analisado. Os dados coletados estiveram muito próximos das características sociodemográficas das comunidades tradicionais ribeirinhas descritas na literatura, como a renda mensal abaixo de um salário mínimo na maioria. No entanto, foram percebidas mudanças no acesso às comunidades, devido aos novos meios de transporte e novas vias terrestre para ligá-la aos centros urbanos, influenciando, dessa forma, a escolaridade, moradia, transporte e atividades econômicas. A população pesquisada, em sua maioria, estava com o esquema vacinal completo de duas doses dos imunizantes contra a COVID-19, conforme as diretrizes de 2022. No entanto, com a publicação das novas diretrizes do Ministério da Saúde em 2024, esse cenário tende a mudar, tendo em vista as peculiaridades encontradas no estudo, como a confiança, o medo das vacinas e o desejo de não se vacinar mais.

Nota-se, ainda, que, na vacinação contra COVID-19, informações sobre as vacinas e fatores sociais podem contribuir de maneira negativa ou positiva. Nessas comunidades, observou-se uma tendência de recusa progressiva das doses da vacina entre os entrevistados, o que pode influenciar o esquema com dose única. O acesso, local e disponibilização da vacina não foram considerados problemas, nem foram relatadas dificuldades para obtê-las. O sentimento em relação à vacina foi positivo para confiança, importância e preocupação, assim como em relação aos processos sociais que inibem ou incentivam a vacinação, mesmo que muitos tenham recebido informações negativas sobre elas.

Considerando as limitações do estudo, pode-se entender que, na vacinação contra a COVID-19 nas comunidades ribeirinhas pesquisadas, houve uma relação positiva entre os fatores sociais e os sentimentos sobre a vacinação contra a COVID-19. Isso ocorreu mesmo diante das comunidades demonstrarem regressão no processo de vacinação. Apesar dos avanços relacionados às características singulares dessa população, nota-se uma carência de informação sobre a importância da vacina. Por fim, sugere-se que os próximos estudos possam ser mais abrangentes em comunidades, a fim de gerar maior acurácia de dados.

## Data Availability

Os dados de pesquisa estão disponíveis no corpo do artigo.
